# Supplementary figures and images for: Spontaneous liver disease in wild-type C57BL/6JOlaHsd mice fed semisynthetic diet
Source: PLoS One. 2020 Sep 21;15(9):e0232069. doi: 10.1371/journal.pone.0232069 (PMC7505464; doi:10.1371/journal.pone.0232069)

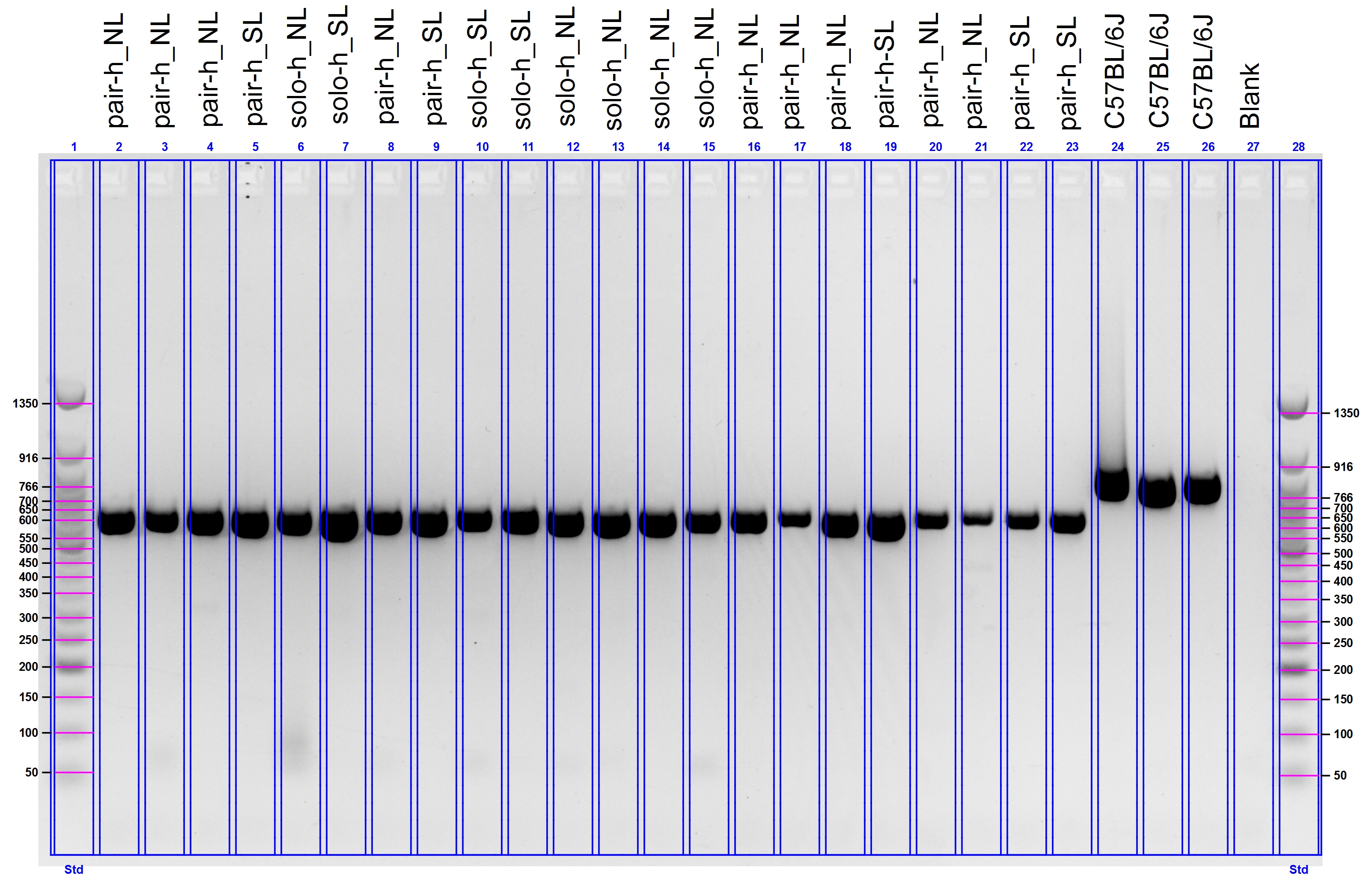

Supplement: S4 Fig — Pair and solo-housed mice dissected at postnatal day (PN) 70 and random C57BL/6J control samples were genotyped for the spontaneous intragenic deletion mutation in the nicotinamide nucleotide transhydrogenase (Nnt) gene. Samples with a band at 743 bp were considered mutant (NntC57BL/6J), whereas samples with a band at 579 bp were considered wild-type (Nnt wild-type). Lane 1 and 28 contain 3 μl DNA ladder (NEB N0556S). Data represent the pair-housed and solo-housed PN70 cohort. Numbers along the ladder indicate fragment length in base pairs. (JPG) [file pone.0232069.s004.jpg]

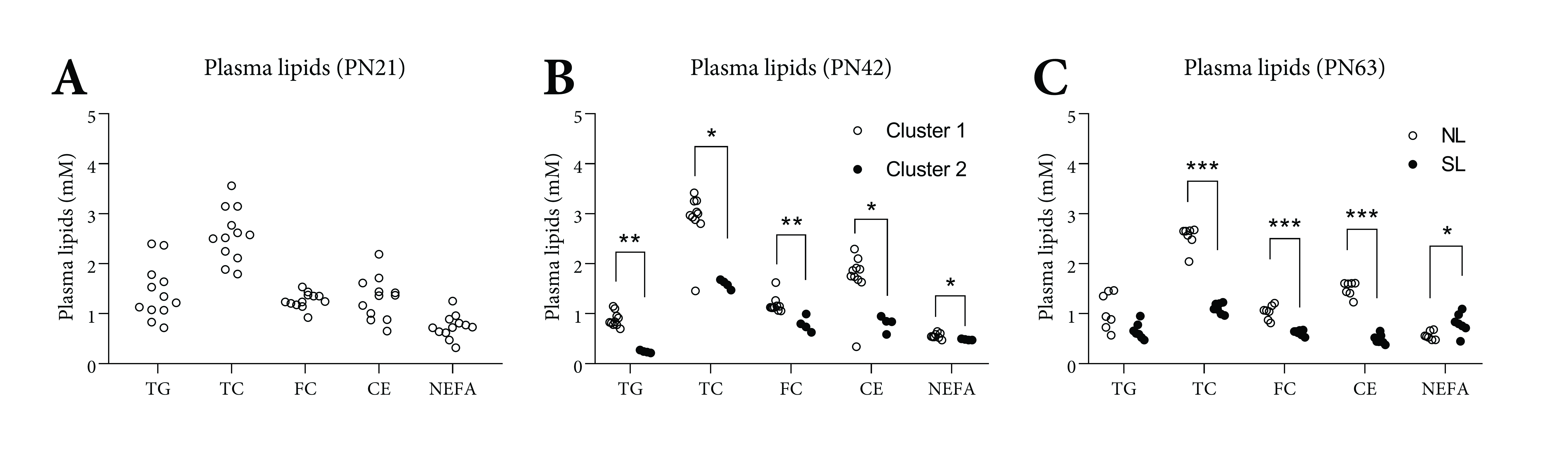

Supplement: S5 Fig — Plasma triglycerides (TG), total (TC), free cholesterol (FC), esterified cholesterol (CE), and non-esterified fatty acids (NEFA) at postnatal day (PN)21 (A), PN42 (B) and PN63 (C). Levels are expressed as absolute concentrations. Mice dissected at PN42 were split into clusters 1 (NL) and 2 (SL) based on principal component analysis (Fig 6F). Data represent the pair-housed PN21 (n = 12 total), PN42 (n = 10 cluster 1, n = 4 cluster 2), and PN63 (n = 7 NL, n = 7 SL) cohorts. Exact two-sided Mann-Whitney U test *: p<0.05, ** p<0.01, *** p<0.001. (TIF) [file pone.0232069.s005.tif]

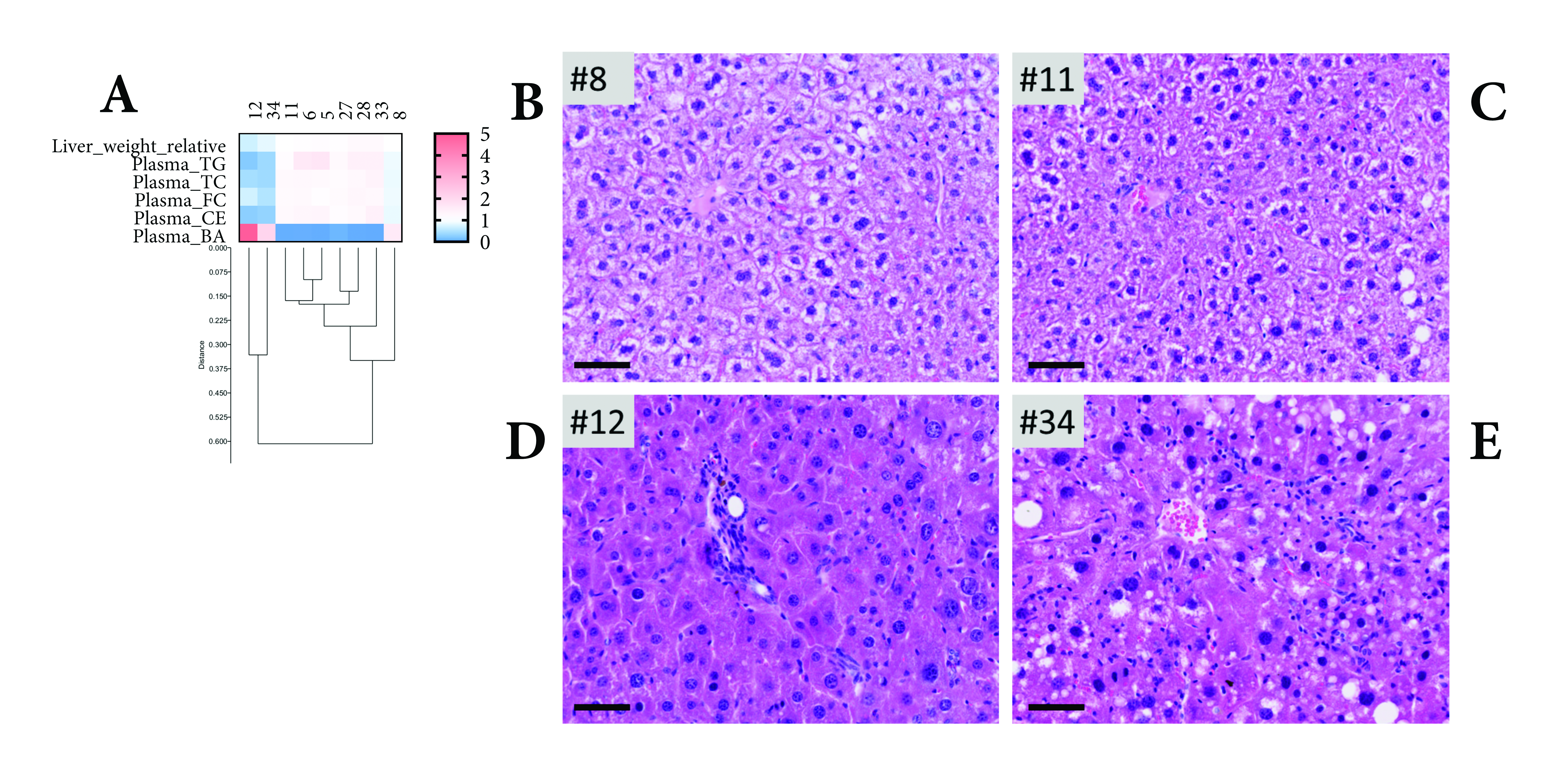

Supplement: S6 Fig — WT C57BL/6J mice, reared on chow, were fed a low-fat semisynthetic control diet (D12450J) from PN56 until PN140. The mice were sacrificed at PN140. Heatmap containing the normalized relative liver weight and plasma lipids and total bile acids. Each column contains data from one mouse. Each row represents a discrete biometric or biochemical parameter. Each square in the heatmap represents a normalized value (parameter value divided by the average of that parameter in all animals). Red color indicates the values is higher than the average, whereas blue color indicates the value is lower than the average of all mice for that particular parameter. Hierarchical clusters were computed using the unweighted pair group method with arithmetic mean (UPGMA) on the Gower's similarity coefficient for mixed data (A). Data represents the pair-housed WT C57BL/6J cohort. Cophenetic correlation coefficient = 0.897. Histological staining using hematoxylin and eosin (‘H&E’) of sample # 8 (B), #11 (C), #12 (D) and #34 (E). Numbers in A correspond to the numbers in B-E. Histology scale bars: 50 μm. (TIF) [file pone.0232069.s006.tif]

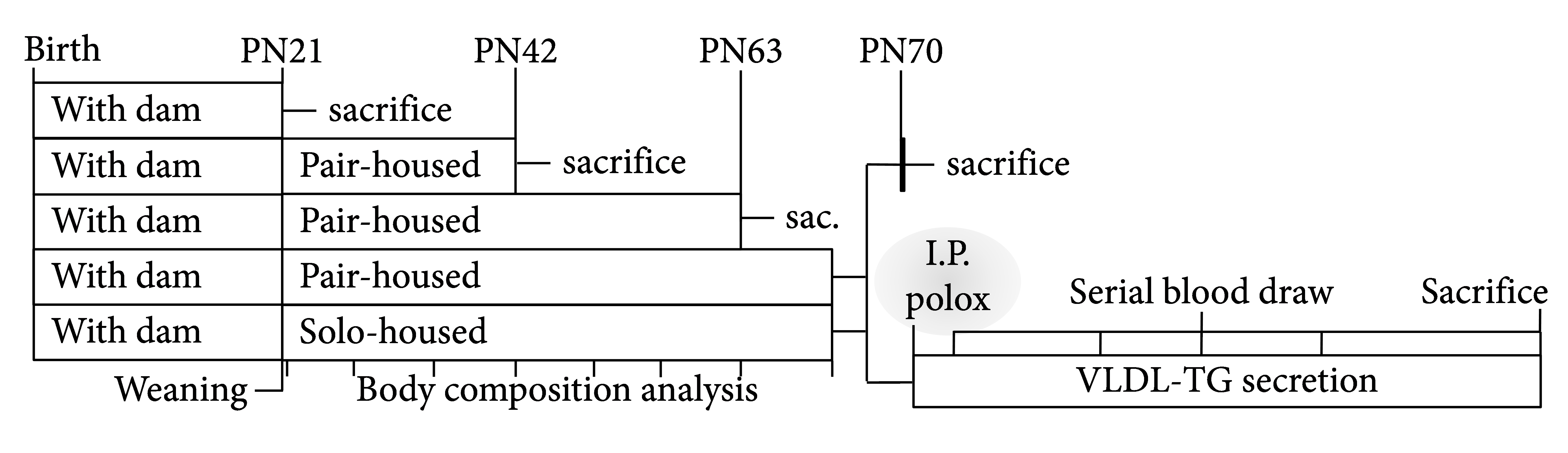

Supplement: S7 Fig — C57BL/6JOlaHsd mice were bred in-house and nests were culled to 4 males and 2 female pups at PN2. Male pups were weaned at PN21 and either pair-housed (entire PN42 cohort, entire PN63 cohort, pair-housed PN70 cohort) or solo-housed (solo-housed PN70 cohort only). Mice were sacrificed at PN21, PN42, PN63 or PN70. In a subset of the PN70 cohort, upon intraperitoneal injection of the lipoprotein lipase inhibitor poloxamer-407, retro-orbital blood was drawn at 0, 1, 2, 3 & 5 h for determining the Very-low density lipoprotein-triglyceride (VLDL-TG) secretion rate. PN21: n = 12, PN42: n = 14, PN63: n = 15, PN70, pair-housed: n = 14, PN70, solo-housed: n = 8, PN70, pair-housed, VLDL-TG experiment: n = 14, PN70, pair-housed, VLDL-TG experiment: n = 8. (TIF) [file pone.0232069.s007.tif]

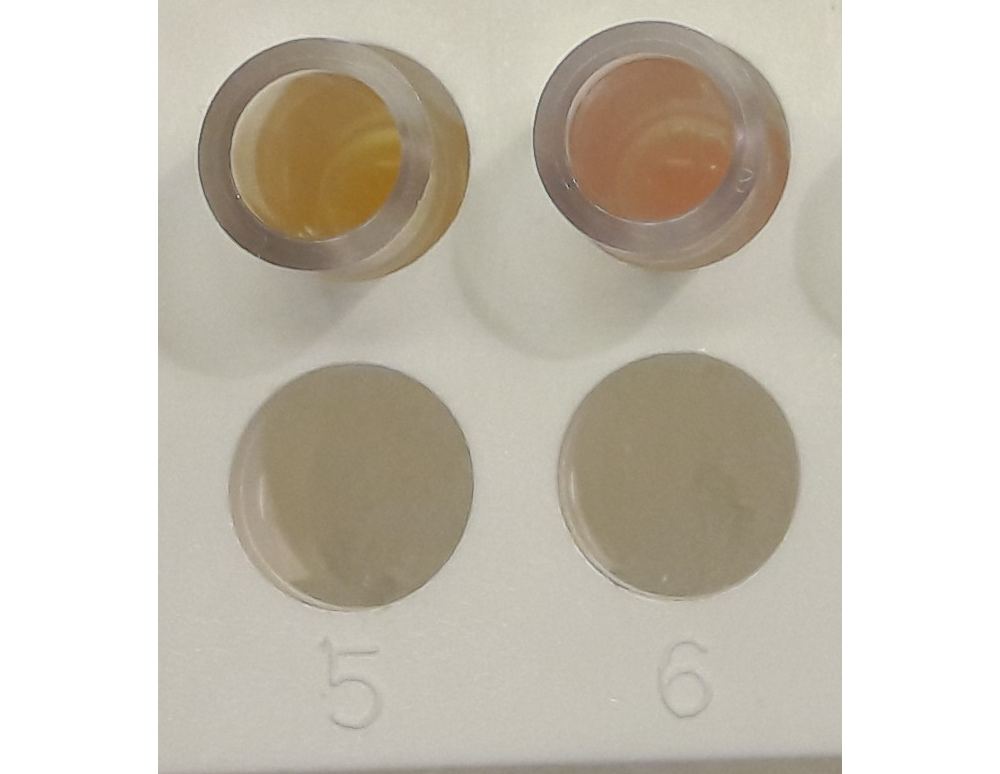

Supplement: S8 Fig — Each tube contained exactly 400 μl of plasma and was not diluted. The left sample (above the ‘5’) was obtained from a mouse which was later labeled “SL”, whereas the right sample (above the ‘6’) was obtained from a mouse which was later labeled “NL”. (TIF) [file pone.0232069.s008.tif]

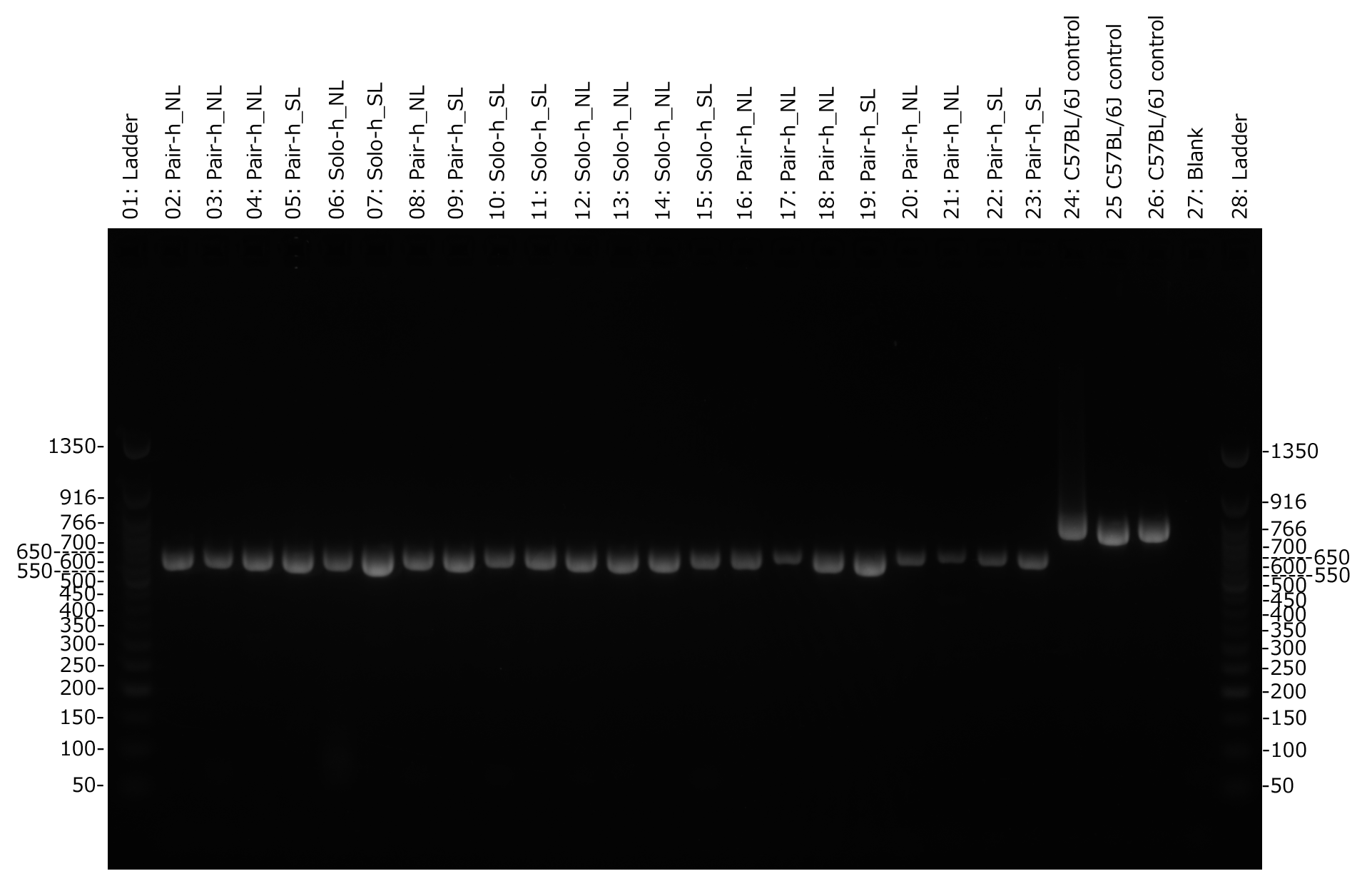

Supplement: S1 Raw image — (TIF) [file pone.0232069.s009.tif]
