# Supplementary material for: Spontaneous liver disease in wild-type C57BL/6JOlaHsd mice fed semisynthetic diet
Source: PLoS One. 2020 Sep 21;15(9):e0232069. doi: 10.1371/journal.pone.0232069 (PMC7505464; doi:10.1371/journal.pone.0232069)
Supplement: S1 Table — Mice dissected at PN42 were split into clusters 1 (NL) and 2 (SL) based on principal component analysis (Fig 6F). Liver (nmol/g) acylcarnitine species are shown as absolute values. Values represent means and SD. Data represent the pair-housed PN42 cohort. Exact two-sided Mann Whitney U test. n.s.: not significantly different between NL and SL. (DOC) [file pone.0232069.s010.doc]

**Supplementary Table 1: Liver acylcarnitine species in SL and NL mice at PN42.** Mice dissected at PN42 were split into clusters 1 (NL) and 2 (SL) based on principal component analysis (Fig. 6F). Liver (nmol/g) acylcarnitine species are shown as absolute values. Values represent means ± SD. Data represent the pair-housed PN42 cohort. Exact two-sided Mann Whitney U test. n.s.: not significantly different between cluster 1 (NL) and cluster 2 (SL).

|  | | Liver (nmol/g) | | | |  |
| --- | --- | --- | --- | --- | --- | --- |
|  | | NL (n=10) | | SL (n=4) | | P-value |
|  |  | mean | SD | mean | SD |  |
| Sum | | 166 | 25 | 244 | 51 | <0.01 |
| Sum C14-C18 | | 1.2 | 2.0 | 7.2 | 7.9 | 0.2 |
| Free/bound ratio | | 3.9 | 1.5 | 2.5 | 1.0 | 0.1 |
| Common name | Abbreviation |  |  |  |  |  |
| L-carnitine | C0 | 128 | 20 | 170 | 49 | 0.1 |
| Acetylcarnitine | C2 | 14 | 12 | 21 | 23 | 0.9 |
| Propionylcarnitine | C3 | 1.05 | 0.66 | 1.40 | 0.81 | 0.3 |
| Butyrylcarnitine | C4 | 0.45 | 0.69 | 0.75 | 0.64 | 0.2 |
| Tiglylcarnitine | C5:1 | 0.07 | 0.00 | 0.07 | 0.00 | 1 |
| Isovaleryl carnitine | C5 | 0.17 | 0.06 | 0.17 | 0.04 | 1 |
| Hexanoylcarnitine | C6 | 0.09 | 0.13 | 0.10 | 0.09 | 0.6 |
| Octanoylcarnitine | C8 | 0.09 | 0.06 | 0.15 | 0.03 | 0.06 |
| Decenoylcarnitine | C10:1 | 0.05 | 0.04 | 0.13 | 0.08 | <0.05 |
| Decanoylcarnitine | C10 | 0.07 | 0.04 | 0.18 | 0.06 | <0.01 |
| Dodecenoylcarnitine | C12:1 | 0.33 | 0.13 | 0.28 | 0.08 | 0.5 |
| Dodecanoylcarnitine | C12 | 0.05 | 0.06 | 0.13 | 0.09 | 0.08 |
| Tetradecenoylcarnitine | C14:1 | 0.06 | 0.04 | 0.18 | 0.16 | 0.08 |
| Tetradecanoylcarnitine | C14 | 0.07 | 0.13 | 0.58 | 0.66 | 0.1 |
| Hexadecenoylcarnitine | C16:1 | 0.15 | 0.30 | 0.90 | 1.06 | 0.5 |
| Hexadecanoylcarnitine | C16 | 0.19 | 0.29 | 2.07 | 2.31 | 0.1 |
| Octadecadienoylcarnitine | C18:2 | 0.05 | 0.08 | 0.23 | 0.27 | 0.4 |
| Octadecenoylcarnitine | C18:1 | 0.57 | 1.11 | 2.65 | 3.03 | 0.5 |
| Octadecanoylcarnitine | C18 | 0.13 | 0.11 | 0.58 | 0.53 | <0.05 |
| Butyrylcarnitine +  Malonylcarnitine | C4OH+C3DC | 2.97 | 0.58 | 3.37 | 0.80 | 0.2 |
| 3-OH-isovalerylcarnitine +  Methylmalonylcarnitine | C5OH+C4DC | 0.57 | 0.08 | 0.77 | 0.09 | <0.01 |
| Glutarylcarnitine | C5DC | 9.1 | 2.7 | 29.1 | 8.3 | <0.01 |
| 3-Methylglutarylcarnitine | C6DC | 0.79 | 0.32 | 3.15 | 0.75 | <0.01 |
