# Supplementary material for: Spontaneous liver disease in wild-type C57BL/6JOlaHsd mice fed semisynthetic diet
Source: PLoS One. 2020 Sep 21;15(9):e0232069. doi: 10.1371/journal.pone.0232069 (PMC7505464; doi:10.1371/journal.pone.0232069)
Supplement: S2 Table — (DOC) [file pone.0232069.s011.doc]

**Supplementary Table 2. Primer and TaqMan probe sequences**

| Gene | NCBI ref. seq. | Forward primer 5’->3’ | Reverse primer 5’->3’ | Taqman probe 5’->3’ |
| --- | --- | --- | --- | --- |
| *36b4* | NM_007475 | GCTTCATTGTGGGAGCAGACA | CATGGTGTTCTTGCCCATCAG | TCCAAGCAGATGCAGCAGATCCGC |
| *Alox15* | NM_009660.3 | GACACTTGGTGGCTGAGGTCTT | TGTAGAGTAGGTGAGGAACTAGAAGCTTAA | CAGGCACCTCATGGTGGCCACA |
| *Atf5* | NM_030693.1 | AGAGCCCCTGGCAGGTGA | CAGAGGAAGGAGAGCTGTGAAGT | ACCCGCTCAGTCATCCAATCAGAGAAGC |
| *Bsep* | NM_021022.3 | CTGCCAAGGATGCTAATGCA | CGATGGCTACCCTTTGCTTCT | TGCCACAGCAATTTGACACCCTAGTTGG |
| *Cd36* | BC010262 | GATCGGAACTGTGGGCTCAT | GGTTCCTTCTTCAAGGACAACTTC | AGAATGCCTCCAAACACAGCCAGGAC |
| *Col1a1* | NM_007742.3 | CGGCTCCTGCTCCTCTTAGG | CTGACTTCAGGGATGTCTTCTTGG | CCACTGCCCTCCTGACGCATGG |
| *Col1a2* | NM_007743.2 | TGTGGATACGCGGACTCTGTT | CTGATCCCGATTGCAAATATTGG | CTGCTTGCAGTAACTTCGTGCCTAGCAACA |
| *Cpt1a* | NM_013495.1 | CTCAGTGGGAGCGACTCTTCA | GGCCTCTGTGGTACACGACAA | CCTGGGGAGGAGACAGACACCATCCAAC |
| *Cyp27a1* | AK004977 | GCCTTGCACAAGGAAGTGACT | CGCAGGGTCTCCTTAATCACA | CCCTTCGGGAAGGTGCCCCAG |
| *Cyp2c70* | NM_145499.2 | ACCAGGGAGATGAGTTTTCTGA | TCCATTGCTGAAGACGATGC | CATCGTTACTCAGTAGAACCAGCCAAGG |
| *Cyp7a1* | NM_007824.2 | CAGGGAGATGCTCTGTGTTCA | AGGCATACATCCCTTCCGTGA | TGCAAAACCTCCAATCTGTCATGAGACCTCC |
| *Cyp8b1* | NM_010012 | AAGGCTGGCTTCCTGAGCTT | AACAGCTCATCGGCCTCATC | CGGCTACACCAAGGACAAGCAGCAAG |
| *Ddit3* | NM_007837.3 | CAGGAAACGAAGAGGAAGAATCA | GCTCCTCTGTCAGCCAAGCTA | CCTTCACTACTCTTGACCCTGCGTCCC |
| *Dgat1* | NM_010046.2 | GGTGCCCTGACAGAGCAGAT | CAGTAAGGCCACAGCTGCTG | CTGCTGCTACATGTGGTTAACCTGGCCA |
| *Dgat2* | NM_026384.2 | GGGTCCAGAAGAAGTTCCAGAAG | CCCAGGTGTCAGAGGAGAAGAG | CCCCTGCATCTTCCATGGCCG |
| *Elovl5* | NM_134255.2 | TGGCTGTTCTTCCAGATTGGA | CCCTTTCTTGTTGTAAGTCTGAATGTA | CATGATTTCCCTGATTGCTCTCTTCACAAAC |
| *Elovl6* | NM_130450.2 | ACACGTAGCGACTCCGAAGAT | AGCGCAGAAAACAGGAAAGACT | TTTCCTGCATCCATTGGATGGCTTC |
| *F4/80* | NM_010130.4 | TCAAGGACACGAGGTTGCTGA | CCAAGGGGCCAATCTGGAA | CCAGCACCCAGGAGCAGCCCA |
| *Fads1* | NM_146094.1 | CCTTCGCGGACATTGTTTACTC | TATGGAGGTCTGCTGCTGCTAT | CTCTGGTTGGACGCTTACCTTCACCA |
| *Fads2* | NM_019699.1 | CCCTGATCGACATTGTGAGTTC | GACGGCAGCTTCATTTATGGA | CCAGCCACAGCTCCCCAGACTTCT |
| *Fas* | NM_007988 | GGCATCATTGGGCACTCCTT | GCTGCAAGCACAGCCTCTCT | CCATCTGCATAGCCACAGGCAACCTC |
| *Foxa2* | NM_010446.1 | GTATGCTGGGAGCCGTGAAG | TTCATGTTGCTCACGGAAGAGTA | CGAGCCATCCGACTGGAGCAGC |
| *Fxr* | NM_009108.2 | GAGGGCTGCAAAGGTTTCTTC | ACTTCCTGCGCATGTACATGTC | CCGTTCTTACACTTGTACACGGCGTTCTTG |
| *Ifn-g* | NM_008337 | GAACTGGCAAAAGGATGGTGAC | GTTGCTGATGGCCTGATTGTC | AGCCAGATTATCTCTTTCTACCTCAGACTCTTTGAAGTC |
| *Il-1b* | NM_008361 | ACCCTGCAGCTGGAGAGTGT | TTGACTTCTATCTTGTTGAAGACAAACC | CCCAAGCAATACCCAAAGAAGAAGATGGAA |
| *Il-6* | NM_031168 | CCGGAGAGGAGACTTCACAGA | AGAATTGCCATTGCACAACTCTT | ACCACTTCACAAGTCGGAGGCTTAATTACA |
| *iNOS* | NM_010927 | CTATCTCCATTCTACTACTACCAGATCGA | CCTGGGCCTCAGCTTCTCAT | CCCTGGAAGACCCACATCTGGCAG |
| *Lxr* | NM_013839 | GCTCTGCTCATTGCCATCAG | TGTTGCAGCCTCTCTACTTGGA | TCTGCAGACCGGCCCAACGTG |
| *Mcp1* | NM_011333.1 | GGCTCAGCCAGATGCAGTTAA | AGCCTACTCATTGGGATCATCTT | CCCCACTCACCTGCTGCTACTCATTCA |
| *Mdr2* | NM_008830.1 | GCAGCGAGAAACGGAACAG | GGTTGCTGATGCTGCCTAGTT | AAAGTCGCCGTCTAGGCGCCGT |
| *Mrp3* | BC046560 | TCCCACTTTTCGGAGACAGTAAC | ACTGAGGACCTTGAAGTCTTGGA | CACCAGTGTCATTCGGGCCTATGGC |
| *Mttp* | NM_008642 | CAAGCTCACGTACTCCACTGAAG | TCATCATCACCATCAGGATTCCT | ACCGCAAGACAGCGTGGGCTACA |
| *Ntcp* | AB003303 | ATGACCACCTGCTCCAGCTT | GCCTTTGTAGGGCACCTTGT | CCTTGGGCATGATGCCTCTCCTC |
| *Pgc1a* | NM_008904 | GACCCCAGAGTCACCAAATGA | GGCCTGCAGTTCCAGAGAGT | CCCCATTTGAGAACAAGACTATTGAGCGAACC |
| *Pgc1b* | NM_133249 | GAGACACAGATGAAGATCCAAGCT | CTTGCCAAGAGAGTCGCTTTGT | CCAGGTGCCTCATGCTGGCCT |
| *Ppara* | NM_011144 | TATTCGGCTGAAGCTGGTGTAC | CTGGCATTTGTTCCGGTTCT | CTGAATCTTGCAGCTCCGATCACACTTG |
| *Pparg* | NM_011146 | CACAATGCCATCAGGTTTGG | GCTGGTCGATATCACTGGAGATC | CCAACAGCTTCTCCTTCTCGGCCTG |
| *Rxr* | NM_011305.3 | GGCAAACATGGGGCTGAAC | GCTTGTCTGCTGCTTGACAGAT | CCAGCTCACCAAATGACCCTGTTACCAAC |
| *Shp* | NM_011850 | AAGGGCACGATCCTCTTCAA | CTGTTGCAGGTGTGCGATGT | ATGTGCCAGGCCTCCGTGCC |
| *Srebp-1c* | NT_039515 | GGAGCCATGGATTGCACATT | CCTGTCTCACCCCCAGCATA | CAGCTCATCAACAACCAAGACAGTGACTTCC |
| *Tgfb1* | NM_011577.1 | GGGCTACCATGCCAACTTCTG | GAGGGCAAGGACCTTGCTGTA | CCCTGCCCCTATATTTGGAGCCTGGAC |
| *Tnfa* | NM_013693 | GTAGCCCACGTCGTAGCAAAC | AGTTGGTTGTCTTTGAGATCCATG | CGCTGGCTCAGCCACTCCAGC |
| *Trib3* | NM_175093.2 | TGTCTTCAGCAACTGTGAGAGGA | CAGTCATCACGCAGGCATC | TCCAGGTTCTCCAGCACCAGCTTC |
| *Xbp1s* | AF443192.1 | GCTGAGTCCGCAGCAGGT | CCCAAAAGGATATCAGACTCAGAATC | CCCAGTTGTCACCTCCCCAGAACATCT |
| *Xbp1u* | NM_013842.2 | ACTATGTGCACCTCTGCAGCA | CCCAAAAGGATATCAGACTCAGAATC | CCCAGTTGTCACCTCCCCAGAACATCT |
| *36b4* | NM_007475.5 | GCTCCAAGCAGATGCAGCA | CCGGATGTGAGGCAGCAG | *(SYBR Green)* |
| *Atf4* | NM_009716.3 | TCCCTTTCCTCTTCCCCTCC | CCGGCTCCGTTATGGC |
| *Atf6* | NM_001081304.1 | GAGAGGTGTCTGTTTCGGGG | CAGCAAACAACGTCGACTCC |
| *Bip* | NM_001163434.1 | GTGTGTGAGACCAGAACCGT | GCAGTCAGGCAGGAGTCTTA |
| *Ctgf* | NM_010217.2 | AAGACACATTTGGCCCAGAC | GACAGGCTTGGCGATTTTAG |
| *Erp72* | NM_009787.2; | ACGCCACCGAACAGACAGAC | CCAGACTGCTCAATCATGTAG |
| *Gadd34* | NM_008654.2 | CCGAGATTCCTCTAAAAGC | CCAGACAGCAAGGAAATGG |
| *Grp94* | NM_011631.1 | CCTGAGAGAACTGATTTCAAATGC | TGTCTGTGACATGCAGCAGG |
| *Hmox1* | NM_010442.2 | AGAATGCTGAGTTCATGAAGAA | CTGCTTGTTGCGCTGTATCTC |
| *Pdi1* | NM_011032.2 | CAAGATCAAGCCCCACCTGAT | CTGCTTGCAGTGACCACACC |
| *Yap1* | NM_009534.3 | GCGGTTGAAACAACAGGAAT | TGCTCCAGTGTAGGCAACTG |
