# Supplementary material for: Spontaneous liver disease in wild-type C57BL/6JOlaHsd mice fed semisynthetic diet
Source: PLoS One. 2020 Sep 21;15(9):e0232069. doi: 10.1371/journal.pone.0232069 (PMC7505464; doi:10.1371/journal.pone.0232069)
Supplement: S3 Table — *: all in FA weight%. (DOC) [file pone.0232069.s012.doc]

**Supplementary Table 3. Fatty acid composition of the AIN-93G diet. *: all in FA weight%**

| Common name | Abbreviation | AIN-93G  D10012G |
| --- | --- | --- |
| **Saturated*** | | **17** |
| Myristic acid | 14:0 | 0.4 |
| Palmitic acid | 16:0 | 12 |
| Stearic acid | 18:0 | 4.1 |
| Arachidic acid | 20:0 | 0.31 |
| Behenic acid | 22:0 | 0.35 |
| Lignoceric acid | 24:0 | 0.14 |
| Cerotic acid | 26:0 | 0.02 |
| **Mono unsaturated*** | | **23** |
| Palmitoleic acid | 16:1ω7 | 0.12 |
| Vaccenic acid | 18:1ω7 | 1.8 |
| Oleic acid | 18:1ω9 | 20 |
| Gondoic acid | 20:1ω9 | 0.27 |
| Erucic acid | 22:1ω9 | - |
| Nervonic acid | 24:1ω9 | - |
| **Polyunsaturated*** | | **61** |
|  | **ω-3 species** | **7.0** |
| α-Linolenic acid | 18:3ω3 | 7.0 |
| Eicosapentaenoic acid | 20:5ω3 | - |
| Docosahexaenoic acid | 22:6ω3 | - |
| Docosapentaenoic acid | 22:5ω3 | - |
|  | **ω-6 species** | **54** |
| Linoleic acid | 18:2ω6 | 54 |
| γ-linolenic acid | 18:3ω6 | - |
| Arachidonic acid | 20:4ω6 | - |
| Dihomo-γ-linolenic acid | 20:3ω6 | - |
| Eicosadienoic acid | 20:2ω6 | 0.11 |
|  | Σ **ω-6 /** Σ **ω-3 ratio** | **7.6** |
| Mead acid | 20:3ω9 | 0.06 |
