# Supplementary material for: Spontaneous liver disease in wild-type C57BL/6JOlaHsd mice fed semisynthetic diet
Source: PLoS One. 2020 Sep 21;15(9):e0232069. doi: 10.1371/journal.pone.0232069 (PMC7505464; doi:10.1371/journal.pone.0232069)
Supplement: S4 Table — (DOC) [file pone.0232069.s013.doc]

**Supplementary Table 4. Calculated nutrient composition (in g/kg) of the diets.**

|  |  | AIN-93G  (D10012G) | LFD  (D12450J) |
| --- | --- | --- | --- |
| Carbohydrate | | 679 | 714 |
| Mono/di-saccharides | | 100 | 69 |
|  | Sucrose | 100 | 69 |
| Polysaccharides | | 529 | 598 |
|  | Maltodextrin | 132 | 118 |
|  | Corn starch | 397 | 480 |
| Fibre |  | 50 | 47 |
|  | Cellulose | 50 | 47 |
| Lipids | | 70 | 43 |
|  | Soybean oil | 70 | 24 |
|  | Lard | - | 19 |
| Protein | | 203 | 193 |
|  | Casein | 200 | 190 |
|  | L-cystine | 3 | 2.8 |
| Mineral mix |  | 35 | 47 |
|  | S10022G | 35 | - |
|  | S10026B | - | 47 |
| Vitamin mix |  | 12.5 | 2.9 |
|  | V10037 | 10 | - |
|  | V10001C | - | 1.0 |
|  | Choline Bitartrate | 2.5 | 1.9 |
| Total | | 1000 | 1000 |
| Total energy | kcal/g (kJ/g) | 3.96 (16.6) | 3.82 (16.0) |
