# Supplementary material for: Spontaneous liver disease in wild-type C57BL/6JOlaHsd mice fed semisynthetic diet
Source: PLoS One. 2020 Sep 21;15(9):e0232069. doi: 10.1371/journal.pone.0232069 (PMC7505464; doi:10.1371/journal.pone.0232069)
Supplement: S5 Table — Mice dissected at PN21 did not show clear clusters, based on principal component analysis (Fig 6F). Liver (nmol/g) acylcarnitine species are shown as absolute values for each mouse. (DOC) [file pone.0232069.s014.doc]

|  | | Liver (nmol/g) | | | | | | | | | | | |
| --- | --- | --- | --- | --- | --- | --- | --- | --- | --- | --- | --- | --- | --- |
| ID | | 01 | 02 | 03 | 04 | 05 | 06 | 07 | 08 | 09 | 10 | 11 | 12 |
| Sum | | 171 | 134 | 123 | 142 | 235 | 251 | 126 | 151 | 259 | 250 | 281 | 148 |
| Sum C14-C18 | | 0.13 | 0.13 | 0.07 | 0.07 | 0.07 | 0.00 | 0.07 | 0.00 | 0.07 | 0.40 | 0.20 | 0.07 |
| Free/bound ratio | | 1.05 | 1.46 | 1.63 | 1.87 | 1.68 | 1.64 | 1.58 | 1.61 | 1.57 | 1.11 | 1.40 | 1.61 |
| Common name | Abbreviation |  |  |  |  |  |  |  |  |  |  |  |  |
| L-carnitine | C0 | 87 | 79 | 77 | 93 | 147 | 156 | 77 | 93 | 158 | 131 | 164 | 91 |
| Acetylcarnitine | C2 | 62.53 | 39.60 | 35.13 | 35.47 | 68.33 | 75.33 | 36.07 | 42.80 | 69.27 | 72.93 | 66.93 | 40.20 |
| Propionylcarnitine | C3 | 1.80 | 0.20 | 0.27 | 0.33 | 0.73 | 0.80 | 0.33 | 0.33 | 0.80 | 1.07 | 1.20 | 0.40 |
| Butyrylcarnitine | C4 | 0.07 | 0.07 | 0.00 | 0.00 | 0.07 | 0.07 | 0.07 | 0.07 | 0.13 | 0.13 | 0.20 | 0.07 |
| Tiglylcarnitine | C5:1 | 0.07 | 0.00 | 0.00 | 0.00 | 0.07 | 0.00 | 0.00 | 0.00 | 0.00 | 0.00 | 0.00 | 0.00 |
| Isovaleryl carnitine | C5 | 0.13 | 0.07 | 0.07 | 0.07 | 0.13 | 0.13 | 0.07 | 0.07 | 0.13 | 0.13 | 0.13 | 0.07 |
| Hexanoylcarnitine | C6 | 0.00 | 0.00 | 0.00 | 0.00 | 0.00 | 0.00 | 0.00 | 0.00 | 0.00 | 0.07 | 0.07 | 0.00 |
| Octanoylcarnitine | C8 | 0.20 | 0.13 | 0.07 | 0.13 | 0.13 | 0.20 | 0.07 | 0.07 | 0.27 | 0.27 | 0.20 | 0.07 |
| Decenoylcarnitine | C10:1 | 0.07 | 0.00 | 0.00 | 0.00 | 0.07 | 0.07 | 0.07 | 0.00 | 0.07 | 0.13 | 0.07 | 0.00 |
| Decanoylcarnitine | C10 | 0.07 | 0.07 | 0.07 | 0.07 | 0.07 | 0.13 | 0.07 | 0.07 | 0.07 | 0.13 | 0.13 | 0.07 |
| Dodecenoylcarnitine | C12:1 | 0.13 | 0.13 | 0.13 | 0.07 | 0.13 | 0.13 | 0.13 | 0.13 | 0.13 | 0.13 | 0.20 | 0.13 |
| Dodecanoylcarnitine | C12 | 0.00 | 0.00 | 0.00 | 0.00 | 0.00 | 0.00 | 0.00 | 0.00 | 0.00 | 0.00 | 0.00 | 0.00 |
| Tetradecenoylcarnitine | C14:1 | 0.00 | 0.00 | 0.00 | 0.00 | 0.00 | 0.00 | 0.07 | 0.00 | 0.00 | 0.00 | 0.00 | 0.00 |
| Tetradecanoylcarnitine | C14 | 0.00 | 0.00 | 0.00 | 0.00 | 0.00 | 0.00 | 0.00 | 0.00 | 0.00 | 0.07 | 0.00 | 0.00 |
| Hexadecenoylcarnitine | C16:1 | 0.00 | 0.00 | 0.00 | 0.00 | 0.00 | 0.00 | 0.00 | 0.00 | 0.00 | 0.00 | 0.00 | 0.00 |
| Hexadecanoylcarnitine | C16 | 0.07 | 0.07 | 0.07 | 0.07 | 0.07 | 0.00 | 0.00 | 0.00 | 0.07 | 0.13 | 0.13 | 0.07 |
| Octadecadienoylcarnitine | C18:2 | 0.00 | 0.00 | 0.00 | 0.00 | 0.00 | 0.00 | 0.00 | 0.00 | 0.00 | 0.00 | 0.00 | 0.00 |
| Octadecenoylcarnitine | C18:1 | 0.00 | 0.00 | 0.00 | 0.00 | 0.00 | 0.00 | 0.00 | 0.00 | 0.00 | 0.07 | 0.00 | 0.00 |
| Octadecanoylcarnitine | C18 | 0.07 | 0.07 | 0.00 | 0.00 | 0.00 | 0.00 | 0.00 | 0.00 | 0.00 | 0.13 | 0.07 | 0.00 |
| Butyrylcarnitine +  Malonylcarnitine | C4OH+C3DC | 2.67 | 2.40 | 2.60 | 2.33 | 2.53 | 2.67 | 1.60 | 2.33 | 3.33 | 4.47 | 8.73 | 2.07 |
| 3-OH-isovalerylcarnitine +  Methylmalonylcarnitine | C5OH+C4DC | 0.60 | 0.47 | 0.40 | 0.47 | 0.73 | 0.93 | 0.47 | 0.53 | 0.80 | 0.73 | 0.53 | 0.33 |
| Glutarylcarnitine | C5DC | 11.13 | 7.87 | 5.27 | 7.67 | 10.87 | 11.00 | 6.40 | 7.60 | 21.60 | 31.33 | 27.07 | 9.53 |
| 3-Methylglutarylcarnitine | C6DC | 1.07 | 0.87 | 0.60 | 0.67 | 1.20 | 1.20 | 0.73 | 0.73 | 1.53 | 3.80 | 7.60 | 1.00 |
